# Prognostic value of echocardiography-estimated right atrial pressure beyond the MAGGIC risk score in heart failure patients

**DOI:** 10.3389/fcvm.2026.1887045

**Published:** 2026-09-23

**Authors:** Dan Wang, Shaohua Wang, Jing Tian, Jingwen Chen, Xingxue Pang

**Affiliations:** 1 Dongzhimen Hospital, Beijing University of Chinese Medicine, Beijing, China; 2 Beijing University of Chinese Medicine, Beijing, China; 3 Peking University First Hospital, Beijing, China; 4 Tianjin University of Traditional Chinese Medicine, Tianjin, China

**Keywords:** all-cause mortality, echocardiography, heart failure, heart failure rehospitalisation, renal venous pressure

## Abstract

**Background:**

Venous congestion plays a central role in the pathophysiology of heart failure (HF). Echocardiography-estimated right atrial pressure (eRAP) provides a readily available non-invasive estimate of right-sided filling pressure and systemic venous congestion. However, it remains unclear whether eRAP can serve as a predictor of all-cause mortality in HF, and whether it can provide prognostic information beyond the scope of existing clinical risk assessments.

**Objective:**

This study investigated the association between eRAP and all-cause mortality in patients hospitalized for HF and evaluated its incremental prognostic value beyond the Meta-Analysis Global Group in Chronic Heart Failure (MAGGIC) risk score.

**Methods:**

This retrospective cohort study included 546 patients hospitalized for HF between January 2021 and December 2024. Patients were categorized as having normal (0–5 mmHg), borderline (5–10 mmHg), or elevated eRAP (10–20 mmHg) according to inferior vena cava (IVC) diameter and inspiratory collapsibility. The primary endpoint was all-cause mortality, with cardiovascular death and HF rehospitalization as secondary endpoints. Cox regression was used to evaluate the association between eRAP and all-cause mortality after adjustment for the MAGGIC risk score. The incremental prognostic value of eRAP beyond MAGGIC was assessed using discrimination, calibration, and decision-curve measures.

**Results:**

During the follow-up period of 1,187.7 person-years, 232 deaths occurred. After adjustment for the MAGGIC risk score, patients in borderline eRAP group and elevated eRAP group had a significantly higher risk of the primary endpoint than those in normal eRAP group (borderline vs. normal: HR 1.47, 95% CI 1.09–1.99, *P* = 0.012; elevated vs. normal: HR 1.69, 95% CI 1.22–2.34, *P* = 0.001). Adding eRAP to the MAGGIC risk score provided modest incremental prognostic value, with improved discrimination (C-index, 0.665 vs. 0.650) and net clinical benefit within selected threshold probability ranges. Elevated eRAP was also associated with cardiovascular death after the MAGGIC risk score adjustment (sHR 1.81, 95% CI 1.26–2.60; *P* = 0.001), whereas no significant association was observed with HF rehospitalization.

**Conclusion:**

eRAP was independently associated with all-cause and cardiovascular mortality in hospitalized patients with HF and provided modest incremental prognostic information beyond the MAGGIC risk score.

## 1 Introduction

Globally, over 64 million individuals are affected by heart failure (HF), a condition associated with a five-year mortality rate exceeding 50% (1, 2). With population ageing and improved survival from cardiovascular diseases, the global burden of HF continues to increase (3). Congestion is a hallmark of HF and an important indicator of disease severity and adverse outcomes (4). Systemic venous congestion, reflected by right atrial pressure (RAP) or central venous pressure (CVP), represents an important hemodynamic feature of HF and is associated with organ dysfunction and poor prognosis (5–7). Therefore, accurate and reliable assessment of systemic venous congestion can provide clinically valuable information for risk stratification in patients with HF. The Venous Excess Ultrasound Score (VExUS) provides a comprehensive approach to assessing systemic venous congestion by integrating inferior vena cava (IVC) assessment with hepatic, portal, and intrarenal venous Doppler patterns (8). This multiparametric approach enables a more detailed characterization of systemic and organ-level venous congestion than assessment based on the IVC alone. However, VExUS requires additional Doppler measurements and technical expertise, and the necessary venous Doppler parameters may not be routinely available in all clinical settings. In contrast, IVC diameter and inspiratory collapsibility are commonly assessed during routine echocardiography and can be used to estimate RAP non-invasively. Thus, eRAP may provide a simpler and more readily available complementary approach to the assessment of systemic venous congestion.

RAP reflects right ventricular filling pressure and systemic venous congestion. However, direct measurement of RAP is invasive and impractical for routine clinical use. Echocardiography is widely used to estimate RAP non-invasively using IVC diameter and collapsibility, and this approach is incorporated into routine cardiovascular assessment (9, 10). Accordingly, the indirect assessment of RAP via echocardiography represents an effective and practical non-invasive clinical strategy. Previous studies have demonstrated associations between abnormal IVC characteristics (11), elevated RAP (12) or CVP (6), and adverse outcomes in patients with HF. However, whether RAP estimated by echocardiography is associated with all-cause mortality in patients with HF and whether it provides incremental prognostic value beyond established clinical risk prediction models remain unclear. In addition, given that renal dysfunction is common in HF (13) and is closely associated with adverse outcomes (14–17), it is also of interest to explore whether renal dysfunction potentially mediates the association between echocardiography-estimated right atrial pressure (eRAP) and long-term prognosis.

The Meta-Analysis Global Group in Chronic Heart Failure (MAGGIC) risk score is a well-established model for predicting all-cause mortality in patients with HF (18). It incorporates multiple established predictors of mortality, including demographic characteristics, clinical parameters, disease severity, renal function, comorbidities, and medical therapy (19). Using the MAGGIC score as the reference model, the study aimed to investigate the association between eRAP and all-cause mortality in patients with HF, and to evaluate whether incorporating eRAP provides incremental prognostic value beyond the MAGGIC risk score. Cardiovascular mortality and HF rehospitalization were additionally evaluated as secondary outcomes, and an exploratory mediation analysis was performed to assess the potential mediating role of renal function.

## 2 Methods

### 2.1 Study design and patient source

A retrospective cohort study was performed comprising patients hospitalized for HF at the Department of Cardiology, Dongzhimen Hospital of Beijing University of Chinese Medicine, between 1 January 2021 and 31 December 2024. Ethical approval for the present investigation was secured from the relevant committee at Dongzhimen Hospital, Beijing University of Chinese Medicine (2025DZMEC-572-02). Owing to the retrospective nature of this study, the requirement for informed consent was waived. The research adhered to the STROBE guidelines and prevailing ethical principles.

### 2.2 Diagnostic criteria

HF was defined as the presence of typical symptoms and/or signs caused by a structural and/or functional cardiac abnormality, supported by elevated natriuretic peptide concentrations and/or objective evidence of pulmonary or systemic congestion. The diagnosis of HF was established by the treating cardiologists and verified through review of the electronic medical records.

### 2.3 Inclusion and exclusion criteria

The inclusion criteria were as follows: hospitalization in the Department of Cardiology, Dongzhimen Hospital, Beijing University of Chinese Medicine, between January 2021 and December 2024; a diagnosis of HF; and availability of echocardiographic measurements required for eRAP estimation, including IVC diameter and inspiratory collapsibility. The exclusion criterion was unavailable follow-up information. A total of 594 patients met the inclusion criteria, of whom 48 were excluded because of loss to follow-up, resulting in a final analytic cohort of 546 patients.

### 2.4 Clinical data and risk assessment

Demographic data, clinical comorbidities, ancillary examination results and medication use were extracted from patients' electronic medical records by trained clinicians. The MAGGIC risk score was calculated for each patient according to the original scoring model developed by Pocock et al. (19), using the clinical variables specified in the model. Although the MAGGIC risk score was originally developed primarily in patients with chronic HF, its prognostic performance has subsequently been externally validated in hospitalized patients with HF (20). Therefore, the MAGGIC risk score was considered appropriate for use in the present hospitalized HF cohort. It was treated as a continuous variable and used as the reference clinical risk measure in the prognostic analyses. Baseline estimated glomerular filtration rate (eGFR) was calculated using the Chronic Kidney Disease Epidemiology Collaboration (CKD-EPI) 2021 equation. Baseline renal insufficiency was defined as eGFR less than 60 mL/min/1.73 m² during hospitalization.

### 2.5 Echocardiographic assessment of eRAP

eRAP was derived from clinically indicated transthoracic echocardiographic examinations performed during the index hospitalization. Because of the retrospective study design, the timing of echocardiography relative to admission and decongestive therapy was not protocolized. According to the recommendations of the American Society of Echocardiography (ASE) (10), eRAP was estimated based on IVC diameter and inspiratory collapsibility as follows: 0–5 mmHg when the IVC diameter was ≤2.1 cm with a collapsibility index ≥50%; 10–20 mmHg when the IVC diameter exceeded 2.1 cm with a collapsibility index <50%; and 5–10 mmHg for measurements falling between these two ranges. Based on eRAP, patients were accordingly divided into three groups: normal eRAP group (0–5 mmHg), borderline eRAP group (5–10 mmHg) and elevated eRAP group (10–20 mmHg).

### 2.6 Outcome measures

All patients were followed up through late March 2026. The primary endpoint was all-cause mortality. Secondary endpoints included cardiovascular death and HF rehospitalization. Survival time was defined as the interval from discharge to death, with survivors censored at the time of last follow-up.

## 3 Statistical analysis

Data are presented as mean (standard deviation) or median (interquartile range) for continuous variables (normality assessed by Shapiro–Wilk) and as numbers (%) for categorical variables. Group comparisons employed analysis of variance (parametric), Kruskal–Wallis (non-parametric), and chi-square/Fisher's exact test (categorical). *Post-hoc* multiple comparisons for variables with statistically significant intergroup differences were conducted using Bonferroni correction.

Kaplan–Meier (KM) methods were employed to generate survival curves for patients with different eRAP levels for all-cause mortality, and differences between groups were evaluated using the log-rank test. Cox proportional hazards regression models were used to evaluate the association between eRAP and all-cause mortality. In the primary analysis, eRAP was modeled as a categorical variable, with normal eRAP as the reference group. Model 0 included the MAGGIC risk score alone. Model 1, the primary model, included eRAP and the MAGGIC risk score. Model discrimination and incremental predictive value were assessed using likelihood-based model comparison, the concordance index (C-index), time-dependent receiver operating characteristic (ROC) analysis, decision curve analysis (DCA), continuous net reclassification improvement (NRI), and integrated discrimination improvement (IDI). The proportional hazards assumption was evaluated using Schoenfeld residual-based tests.

For secondary endpoints, cardiovascular death and HF rehospitalization were analyzed as competing risk outcomes. Cumulative incidence function (CIF) curves were generated by eRAP group and compared using Gray's test. Multivariable Fine-Gray competing risk models were used to estimate subdistribution hazard ratios. Prediction performance for competing risk models was evaluated using time-dependent area under the curve (AUC) and Brier score.

Subgroup analyses explored differences in overall effects across distinct patient characteristics. Sensitivity analyses were conducted to assess the robustness of the primary findings. Model 2 treated eRAP as an ordinal variable. Model 3 further adjusted for B-type natriuretic peptide (BNP). Model 4 additionally adjusted for early diastolic mitral inflow velocity to mitral annular tissue velocity (E/e') ratio and main pulmonary artery (MPA) diameter. The primary model was also repeated after excluding patients who died during hospitalization and compared with Model 0 fitted in the same restricted sample.

Internal validation of the primary model was performed using bootstrap resampling with 1,000 samples drawn with replacement from the original cohort. Exploratory mediation analysis was conducted to evaluate whether eGFR mediated the association between eRAP and all-cause mortality. Linear regression was used for the mediator model, Cox regression was used for the outcome model, and mediation effects were estimated on the log hazard ratio scale with bootstrap confidence intervals. Hazard ratios (HRs) with 95% confidence intervals (CIs) were reported for Cox models; subdistribution hazard ratios (95% CI) for Fine-Gray models. All tests were two-tailed with significance at *p* < 0.05. Data analysis and visualization were performed using Python (version 3.9.13) and R (version 4.4.3).

## 4 Results

### 4.1 Baseline characteristics and clinical outcomes

We enrolled 594 patients at the start of the study. Following the exclusion of 48 cases due to loss to follow-up, the final analysis cohort comprised 546 patients. The study population (median age 77 years) consisted of 265 male patients, comprising 48.5% of the cohort. With a median left ventricular ejection fraction (LVEF) of 56%, the cohort predominantly comprised patients with heart failure with preserved ejection fraction, accounting for 63.4% of the study population. Among these, 67.9% had concomitant coronary artery disease (CAD), 71.6% had a history of hypertension, 44.1% had diabetes mellitus, and 50.6% of patients had eGFR less than 60 mL/min/1.73 m². During 1,187.7 person-years of follow-up, 232 all-cause deaths, 324 HF rehospitalizations, and 185 cardiovascular deaths were observed. Intergroup comparisons revealed statistically significant differences among the three groups in HF duration, blood pressure, heart rate, LVEF, E/e' ratio, MPA diameter, BNP levels (Kruskal–Wallis test), and hypertension classification (chi-square test) (Table 1). Patients with elevated eRAP tended to exhibit a more adverse haemodynamic and echocardiographic profile, characterized by lower LVEF, higher E/e′ ratios and BNP levels. Significant disparities in the rates of all-cause mortality across the groups were initially identified using chi-square tests (Table 1). Compared with the normal eRAP group, both the borderline eRAP group and the elevated eRAP group exhibited significantly increased rates of all-cause mortality (normal vs. borderline, *P* = 0.012; normal vs. elevated, *P* = 0.007). Furthermore, statistically significant differences in cardiovascular death were observed between the normal and elevated groups (*P* = 0.007) (Supplementary Table S1).

**Table 1:** Baseline clinical characteristics and follow-up outcomes of patients according to eRAP.

Variables	Total (*n* = 546)	Normal (*n* = 265)	Borderline (*n* = 162)	Elevated (*n* = 119)	*P*
Demographic and Clinical Characteristics					
Age (years)	77.00 (69.25–83.00)	78.00 (70.00–84.00)	77.00 (71.00–83.00)	75.00 (67.50–81.50)	0.242
Male, *n* (%)	265 (48.53)	129 (48.68)	69 (42.59)	67 (56.30)	0.076
BMI, (kg/m²)	24.15 (21.00–27.06)	24.01 (21.00–27.04)	24.22 (21.00–27.70)	23.89 (21.00–27.14)	0.870
HF duration <18 months, *n* (%)	334 (61.17)	178 (67.17)	92 (56.79)	64 (53.78)	0.018
Length of hospital stay (days)	9.00 (6.00–13.00)	9.00 (7.00–12.00)	8.00 (6.00–12.00)	8.00 (6.00–14.00)	0.485
Smoking, *n* (%)	152 (27.84)	79 (29.81)	36 (22.22)	37 (31.09)	0.158
Alcohol consumption, *n* (%)	116 (21.25)	60 (22.64)	28 (17.28)	28 (23.53)	0.333
Vital Signs					
SBP (mmHg)	132.00 (99.00–152.00)	136.00 (110.00–154.00)	127.00 (99.00–152.75)	126.00 (99.00–147.00)	0.001
DBP (mmHg)	80.00 (68.00–94.00)	76.00 (65.00–90.00)	82.00 (69.00–95.00)	84.00 (71.00–96.50)	0.021
HR (beats per minute)	86.00 (72.00–99.00)	85.00 (71.00–98.00)	89.50 (76.00–99.00)	85.00 (74.50–98.00)	0.021
Respiratory rate (breaths/min)	20.00 (18.00–20.00)	19.00 (18.00–20.00)	20.00 (18.00–20.00)	20.00 (18.00–20.00)	0.178
Laboratory Parameters and Clinical Scores					
Serum Creatinine	95.00 (74.00–130.75)	96.00 (74.00–130.00)	92.00 (70.00–127.75)	97.00 (78.00–134.50)	0.387
eGFR	59.53 (39.31–80.02)	59.22 (39.31–77.65)	60.25 (42.37–82.93)	56.93 (36.64–79.75)	0.545
BNP	647.50 (285.00–1,812.69)	551.00 (263.00–1,229.18)	725.25 (299.86–2,006.72)	1,062.00 (383.76–2,448.22)	<0.001
LVEF	56.00 (42.00–64.00)	58.00 (46.00–64.00)	56.00 (40.00–64.00)	51.00 (38.50–61.00)	0.012
LVEF Class, *n* (%)					0.012
HFrEF	129 (23.63)	50 (18.87)	44 (27.16)	35 (29.41)	
HFmrEF	71 (13.00)	28 (10.57)	22 (13.58)	21 (17.65)	
HFpEF	346 (63.37)	187 (70.57)	96 (59.26)	63 (52.94)	
E/e' ratio	16.90 (12.40–21.38)	16.50 (12.20–19.90)	16.00 (11.75–21.82)	18.80 (14.30–23.00)	0.022
MPA Diameter	25.00 (22.00–27.00)	24.00 (22.00–26.00)	25.00 (23.00–27.00)	26.00 (24.00–29.00)	<0.001
MAGGIC risk score	30.00 (25.00–33.75)	30.00 (25.00–33.00)	30.00 (26.00–33.00)	30.00 (26.00–34.00)	0.570
NYHA classification, *n* (%)					0.214
Class I	1 (0.18)	1 (0.38)	0 (0.00)	0 (0.00)	
Class II	14 (2.56)	7 (2.64)	6 (3.70)	1 (0.84)	
Class III	130 (23.81)	74 (27.92)	32 (19.75)	24 (20.17)	
Class IV	401 (73.44)	183 (69.06)	124 (76.54)	94 (78.99)	
Medical History and Comorbidities, *n* (%)					
Hypertension					0.011
None	155 (28.39)	60 (22.64)	47 (29.01)	48 (40.34)	
Grade 1	23 (4.21)	12 (4.53)	10 (6.17)	1 (0.84)	
Grade 2	87 (15.93)	45 (16.98)	24 (14.81)	18 (15.13)	
Grade 3	281 (51.47)	148 (55.85)	81 (50.00)	52 (43.70)	
CAD	371 (67.95)	182 (68.68)	112 (69.14)	77 (64.71)	0.689
Renal Insufficiency	276 (50.55)	135 (50.94)	79 (48.77)	62 (52.10)	0.845
COPD	59 (10.81)	32 (12.08)	15 (9.26)	12 (10.08)	0.635
Diabetes mellitus	241 (44.14)	111 (41.89)	75 (46.30)	55 (46.22)	0.589
Medication Use, *n* (%)					
Beta-blockers	373 (68.32)	186 (70.19)	115 (70.99)	72 (60.50)	0.115
ACEIs/ARBs	122 (22.34)	68 (25.66)	34 (20.99)	20 (16.81)	0.138
Survival Prognosis, *n* (%)					
All-Cause Mortality	232 (42.49)	92 (34.72)	79 (48.77)	61 (51.26)	0.002
CV Death	185 (33.88)	74 (27.92)	59 (36.42)	52 (43.70)	0.008
HF Rehospitalization	324 (59.34)	153 (57.74)	96 (59.26)	75 (63.03)	0.621

BMI, body mass index; SBP, systolic blood pressure; DBP, diastolic blood pressure; HR, heart rate; HF, heart failure; eGFR, estimated glomerular filtration rate; LVEF, left ventricular ejection fraction; HFrEF, heart failure with reduced ejection fraction; HFmrEF, heart failure with mildly reduced ejection fraction; HFpEF, heart failure with preserved ejection fraction; E/e' ratio, early diastolic mitral inflow velocity to mitral annular tissue velocity ratio; MPA diameter, main pulmonary artery diameter; BNP, B-type natriuretic peptide; MAGGIC, Meta-Analysis Global Group in Chronic Heart Failure; NYHA, New York Heart Association; CAD, coronary artery disease; COPD, chronic obstructive pulmonary disease; ACEIs, angiotensin-converting enzyme inhibitors; ARBs, angiotensin II receptor blockers; CV death, cardiovascular death.

### 4.2 Analysis of the correlation between eRAP and all-cause mortality

The frequencies of the all-cause mortality among the three patient groups were as follows: the normal group 34.7% (92/265), the borderline eRAP group 48.8% (79/162), and the elevated eRAP group 51.3% (61/119). Elevated eRAP was associated with a markedly higher probability of all-cause mortality (*P* = 0.001), as illustrated by Kaplan–Meier curves (Figure 1). Two analytical approaches were employed: univariate Cox regression, which included only eRAP, and Model 1, which additionally incorporated the MAGGIC score along with eRAP. In univariate Cox regression analysis, eRAP exhibited a significant positive relationship with all-cause mortality (overall *P* = 0.001). Compared with patients with the normal eRAP, those with borderline eRAP had a 51% higher risk of all-cause mortality (HR 1.51, 95% CI 1.12–2.05; *P* = 0.007), and those with elevated eRAP had a 74% higher risk (HR 1.74, 95% CI 1.26–2.41; *P* < 0.001). The robustness of this association was maintained after controlling for MAGGIC risk score (overall *P* = 0.003) (Model 1). Compared with the normal eRAP group, the borderline eRAP group remained associated with a higher risk of mortality (adjusted HR 1.47, 95% CI 1.09–1.99; *P* = 0.012), as did the elevated eRAP group (adjusted HR 1.69, 95% CI 1.22–2.34; *P* = 0.001). The MAGGIC risk score was also independently associated with mortality (HR per 1-point increase 1.11, 95% CI 1.08–1.13; *P* < 0.001) (Table 2). No evidence of violation of the proportional hazards assumption was observed for the primary Cox model. The results of proportional hazards assumption testing for all Cox regression models are provided in supplementary file (Supplementary Table S2).

**Figure 1:**
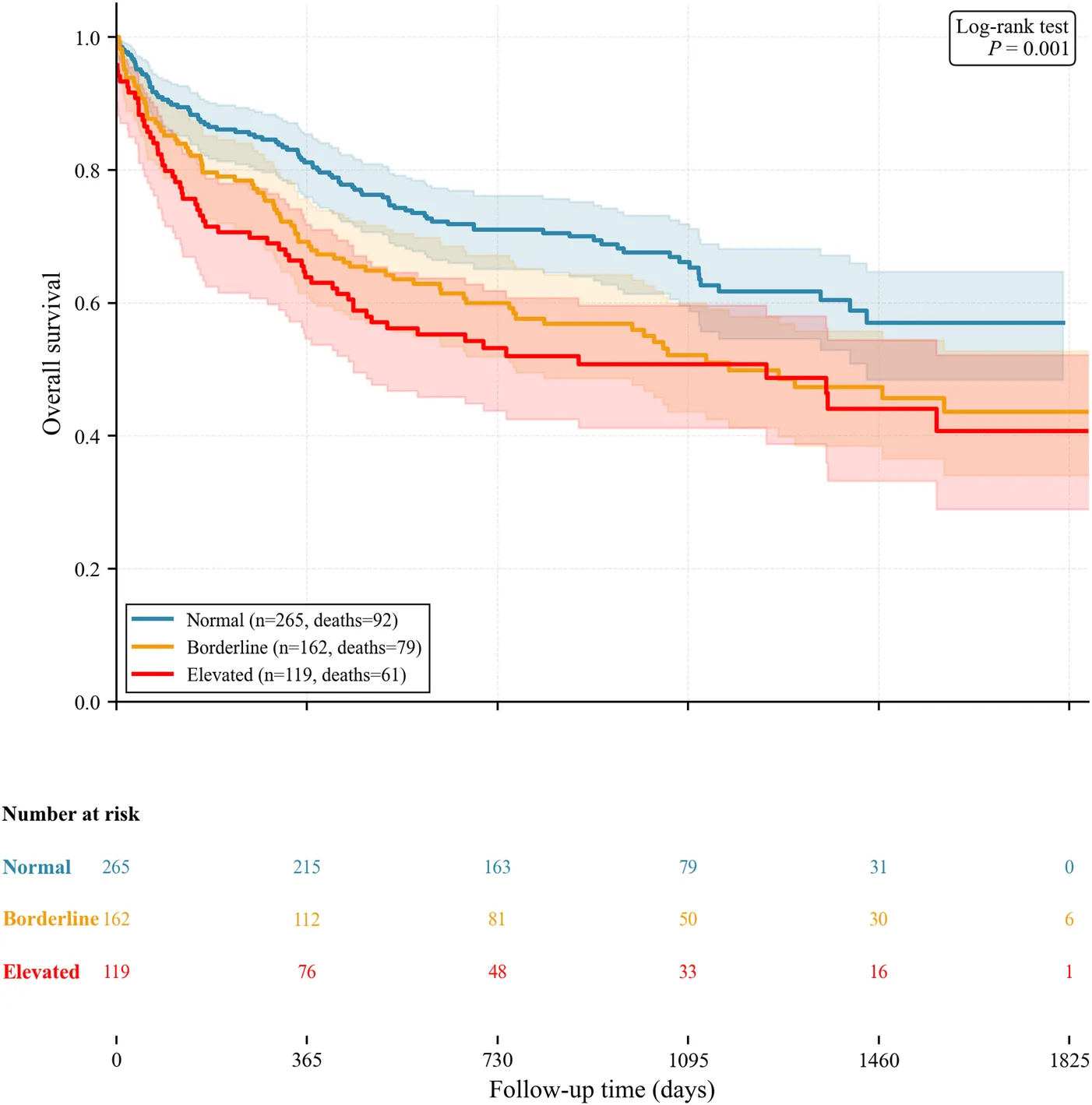
Kaplan–meier survival curves according to eRAP. eRAP, echocardiography-estimated Right Atrial Pressure.

**Table 2:** Cox regression models for the association between eRAP and all-cause mortality in HF patients.

Model	Variables	HR(95%CI)	*P*	C-Index
Univariate COX	eRAP		0.001	0.572
	normal eRAP	Reference		
	borderline eRAP	1.51 (1.12–2.05)	0.007	
	elevated eRAP	1.74 (1.26–2.41)	<0.001	
Model 1^a^	eRAP		0.003	0.665
	normal eRAP	Reference		
	borderline eRAP	1.47 (1.09–1.99)	0.012	
	elevated eRAP	1.69 (1.22–2.34)	0.001	
	MAGGIC risk score	1.11 (1.08–1.13)	<0.001	

eRAP, echocardiography-estimated right atrial pressure; HR, hazard ratio; CI, confidence interval; C-index, concordance index; MAGGIC, Meta-Analysis Global Group in Chronic Heart Failure.

a echocardiography-estimated Right Atrial Pressure, MAGGIC Risk Score.

### 4.3 The predictive value of eRAP beyond the MAGGIC score

To determine whether eRAP added incremental predictive value beyond the MAGGIC score, Model 0 was constructed to include only the MAGGIC risk score. Model 1 incorporated eRAP on top of Model 0. The addition of eRAP provided modest incremental prognostic value beyond the MAGGIC risk score for all-cause mortality in patients with HF (Table 3). This finding was corroborated by C-index, Net Reclassification Improvement (NRI), and Integrated Discrimination Improvement (IDI) metrics (Table 4). Additionally, the time-dependent AUC showed a progressive increase (Supplementary Figure S1). Finally, DCA was performed to determine the potential clinical benefit of the models. DCA suggested that Model 1 generally achieved higher net benefit than Model 0 across the selected threshold probability range, indicating potential incremental clinical net benefit from incorporating eRAP into the MAGGIC-based prediction model (Figure 2).

**Table 3:** Model Fit and discrimination of Cox proportional hazards models.

Model	−2 log-likelihood	AIC	BIC	C-Index	LR *χ*²	*P*
Model 0^a^	2,676.61	2,678.61	2,682.92	0.650	Reference	Reference
Model 1^b^	2,664.73	2,670.73	2,683.64	0.665	11.88	0.003

eRAP, echocardiography-estimated right atrial pressure; HR, hazard ratio; CI, confidence interval; C-index, concordance index; MAGGIC, Meta-Analysis Global Group in Chronic Heart Failure.

a MAGGIC Risk Score.

b echocardiography-estimated Right Atrial Pressure, MAGGIC Risk Score.

**Table 4:** Added predictive ability and reclassification statistics beyond the MAGGIC score.

Time	Model	metric	Estimate (95% CI)	P	NRI	IDI	N
6-month	Model 1	Continuous NRI	0.335 (0.119–0.561)	0.004	0.335		546
		IDI	0.014 (0.002–0.039)	0.008		0.014	546
1-year	Model 1	Continuous NRI	0.368 (0.169–0.565)	<0.001	0.368		546
		IDI	0.019 (0.004–0.045)	0.002		0.019	546
2-year	Model 1	Continuous NRI	0.341 (0.160–0.524)	<0.001	0.341		486
		IDI	0.020 (0.004–0.050)	<0.001		0.020	486

CI, confidence interval; NRI, net reclassification improvement; IDI, integrated discrimination improvement; MAGGIC, Meta-Analysis Global Group in Chronic Heart Failure.

Model 1: echocardiography-estimated Right Atrial Pressure, MAGGIC Risk Score.

**Figure 2:**
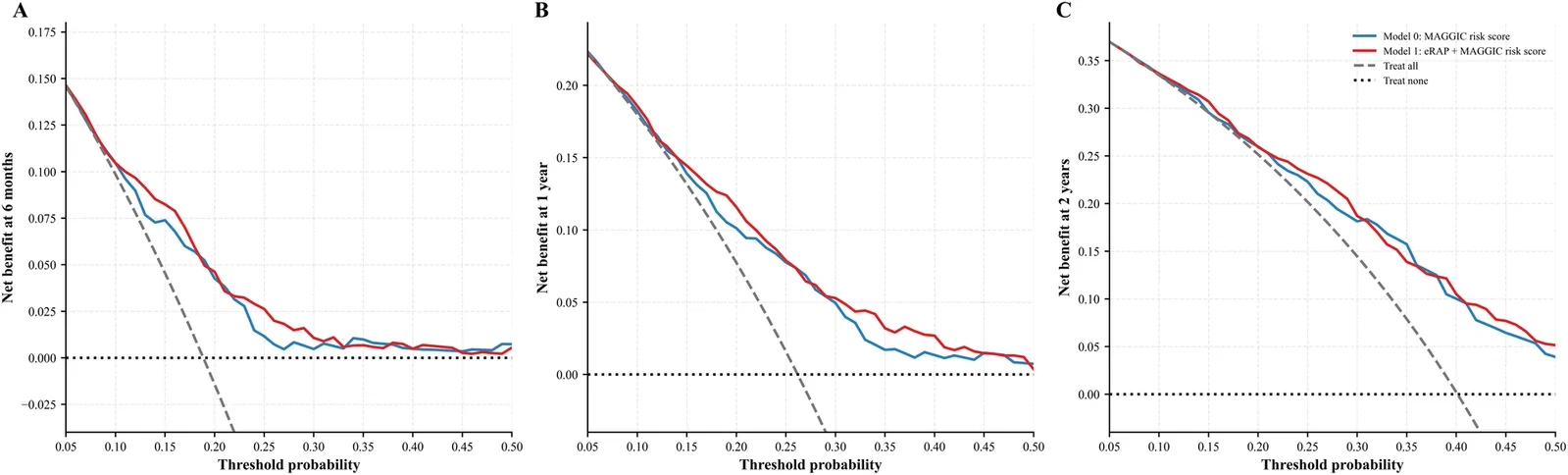
Decision curve analysis. **(A)** 6 months, **(B)** 1 year, and **(C)** 2 years. MAGGIC Meta-Analysis Global Group in Chronic Heart Failure. eRAP, echocardiography-estimated Right Atrial Pressure.

### 4.4 Subgroup analysis

Subgroup analyses were conducted according to age, cardiac function classification, renal insufficiency, and LVEF. The association linking eRAP to risk of all-cause mortality remained consistent in direction across all subgroups. Although point estimates varied across subgroups, no significant interactions were observed (Figure 3).

**Figure 3:**
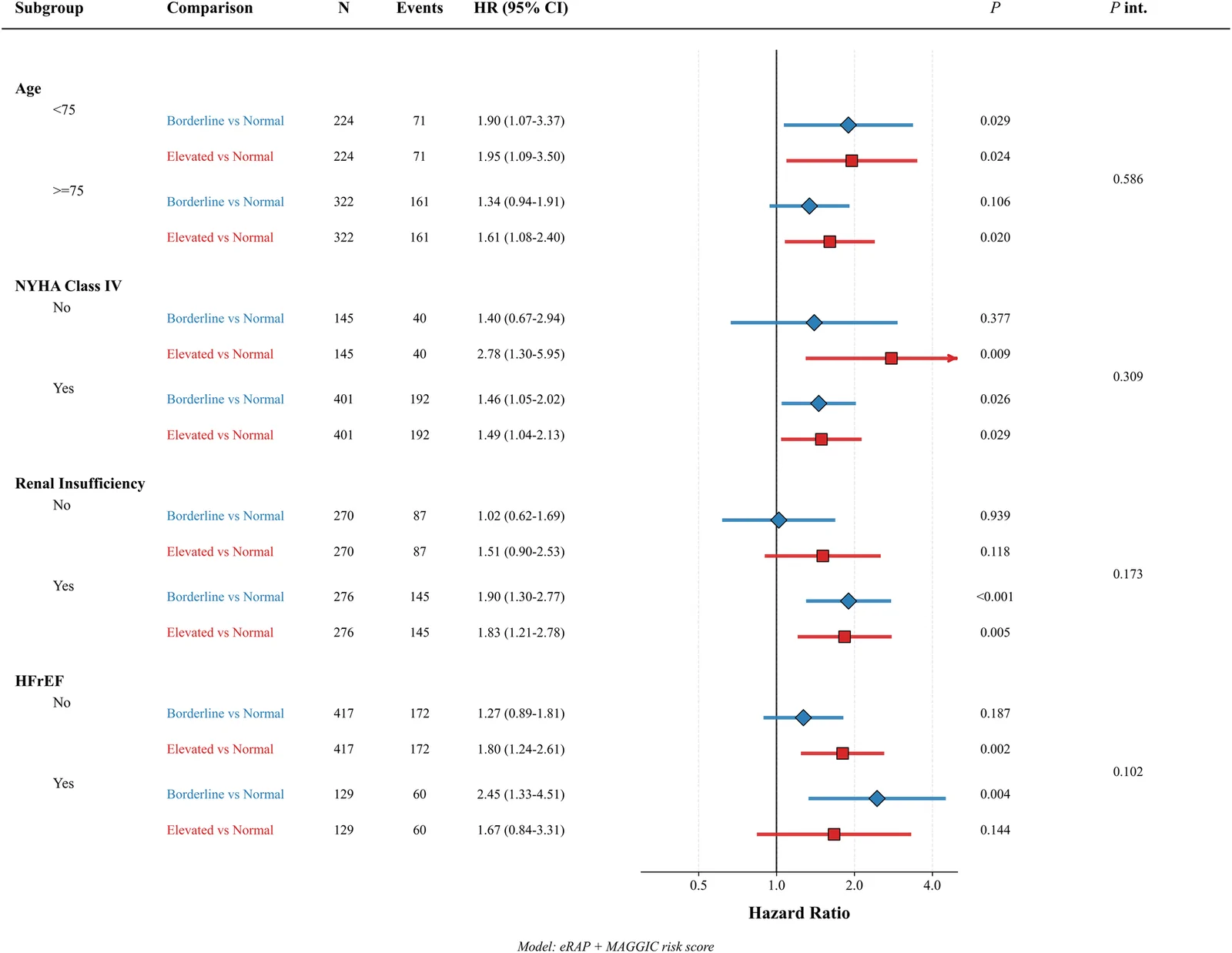
Subgroup analyses stratified of the association between eRAP and all-cause mortality. NYHA, New York Heart Association functional class IV; HFrEF, heart failure with reduced ejection fraction; MAGGIC, Meta-Analysis Global Group in Chronic Heart Failure; eRAP, echocardiography-estimated right atrial pressure; HR, hazard ratio; CI, confidence interval.

### 4.5 Secondary endpoints

CIF analysis demonstrated significant differences in cardiovascular death across eRAP categories (Gray's test, *P* = 0.004), whereas no significant difference was observed for HF rehospitalization (Gray's test, *P* = 0.472) (Supplementary Figure S2). In the Fine–Gray competing-risk regression, the elevated eRAP group was associated with a higher risk of cardiovascular death compared with the normal eRAP group in the unadjusted model (sHR 1.82, 95% CI 1.27–2.61; *P* = 0.001), and the association remained essentially unchanged after adjustment for the MAGGIC risk score (sHR 1.81, 95% CI 1.26–2.60; *P* = 0.001). By contrast, eRAP was not significantly associated with HF rehospitalization in either model (Supplementary Table S3). Furthermore, adding eRAP to the MAGGIC-based model improved prediction of cardiovascular death at 6 months, 1 year, and 2 years, as indicated by higher time-dependent AUCs and lower Brier scores, whereas no material improvement was observed for HF rehospitalization (Supplementary Table S4).

### 4.6 Sensitivity analysis

Sensitivity analyses showed results consistent with the primary analysis. When eRAP was modeled as an ordinal variable, each higher eRAP category was significantly associated with an increased risk of all-cause mortality (HR 1.31, 95% CI 1.12–1.54, *P* < 0.001). After additional adjustment for BNP, borderline and elevated eRAP remained significantly associated with increased mortality risk. In the model additionally adjusted for E/e′ ratio and MPA diameter, elevated eRAP remained significant, while borderline eRAP showed a marginal association. After excluding six patients who died during hospitalization, both borderline and elevated eRAP remained significantly associated with all-cause mortality (Supplementary Table S5). All sensitivity models improved model fit compared with the corresponding MAGGIC-only model, supporting the robustness of the primary findings (Supplementary Table S6).

### 4.7 Exploratory mediation analysis of baseline eGFR

Exploratory mediation analysis was performed to assess whether baseline eGFR potentially mediated the association between eRAP and all-cause mortality. Although lower baseline eGFR was associated with a higher risk of all-cause mortality, no significant association was observed between eRAP and baseline eGFR (Supplementary Table S8). In the fully adjusted model, the indirect effect through baseline eGFR was not significant for either borderline eRAP (indirect effect, −0.041; 95% CI, −0.117–0.028; *P* = 0.284) or elevated eRAP (indirect effect, −0.002; 95% CI, −0.081–0.072; *P* = 0.960) (Supplementary Table S9). These findings provided no evidence that baseline eGFR significantly mediated the association between eRAP and all-cause mortality.

### 4.8 Bootstrap internal validation

Bootstrap internal validation with 1,000 resamples showed minimal optimism, with the C-index decreasing from 0.665 to 0.661 and the calibration slope from 1.000 to 0.977 after optimism correction (Supplementary Table S7). The associations between eRAP and all-cause mortality remained stable across bootstrap resamples, supporting the robustness and internal validity of the primary model.

## 5 Discussion

In this study, higher eRAP was associated with an increased risk of all-cause mortality in patients with HF and this association persisted after adjustment for the MAGGIC risk score. Adding eRAP to the MAGGIC risk score provided modest incremental prognostic value, as evidenced by improved model discrimination and clinical net benefit. Both findings remained robust in sensitivity analyses and bootstrap internal validation. Subgroup analyses showed generally consistent associations between higher eRAP and increased all-cause mortality across clinically relevant subgroups, with no significant interaction observed, further supporting the robustness of the primary findings. Regarding secondary outcomes, higher eRAP was associated with cardiovascular mortality but not with HF rehospitalization. In addition, exploratory mediation analysis showed no significant mediation of the association between eRAP and all-cause mortality by baseline eGFR.

RAP reflects right-sided filling pressure and the degree of systemic venous congestion and may provide an important window into the overall hemodynamic status of HF. Traditionally, the hemodynamic consequences of HF have been viewed primarily in terms of reduced cardiac output and impaired forward organ perfusion (21). However, in recent years, the role of venous congestion and elevated venous pressures in multiorgan dysfunction has gained increasing attention (22). From a hemodynamic perspective, effective organ perfusion depends not only on arterial pressure but also on downstream venous pressure. As RAP rises and elevated venous pressure is transmitted to peripheral organs, the arteriovenous perfusion gradient may decrease while tissue congestion increases, potentially contributing to multiorgan dysfunction. For example, in the kidney, renal venous congestion and reduced renal perfusion pressure may contribute to renal dysfunction (23, 24); in the liver, hepatic venous congestion may lead to hepatocellular injury and the development of hepatic fibrosis (25); in the gut, intestinal venous congestion may impair nutrient absorption, disrupt intestinal barrier integrity, and promote endotoxin translocation and systemic inflammation (26). At the cellular and molecular level, venous congestion may promote a shift in the endothelial phenotype toward pro-inflammatory, pro-oxidative, and vasoconstrictive states, thereby contributing to endothelial dysfunction, microcirculatory impairment, and subsequent tissue injury (27). Therefore, elevated RAP may reflect a more advanced hemodynamic phenotype characterized by systemic venous congestion and impaired organ perfusion, providing a plausible pathophysiological context for its association with increased all-cause mortality.

The addition of eRAP improved the prognostic performance of the MAGGIC score, suggesting that the right atrial filling pressure and systemic venous congestion reflected by eRAP may capture a complementary dimension of risk that is not fully represented by conventional clinical risk factors, thereby refining risk stratification. Sensitivity analyses incorporating additional markers of cardiac dysfunction and congestion, including the E/e′ ratio and pulmonary artery diameter, further supported this interpretation. Hence, eRAP may serve as a complementary marker to modestly optimize mortality risk assessment. Non-invasive methods for estimating RAP are currently available in clinical practice. One such method is ultrasound measurement of jugular venous pressure (28). Others include compression ultrasound of forearm venous pressure (29) and assessment of IVC diameter and collapsibility index (9). Among these, data on IVC diameter and IVC collapsibility index can be readily obtained during echocardiography. Moreover, echocardiography is already a routine examination during hospitalisation for HF patients (30, 31). Consequently, IVC-based estimation of RAP can be readily incorporated into routine echocardiographic assessment without requiring additional specialized testing, supporting its feasibility as a complementary prognostic marker in patients with HF.

In this study, we did not observe a significant association between eRAP and HF rehospitalization, which differs from some previous reports (6, 32). This discrepancy may be attributable to the dynamic nature of venous congestion, as a single measurement of eRAP during hospitalization may not adequately capture residual congestion at discharge, a potentially more relevant determinant of subsequent HF rehospitalization. Moreover, HF rehospitalization is influenced by multiple factors, including treatment adjustments, medication adherence (33), and outpatient management. In addition, patients with higher eRAP in our cohort had a lower prevalence of a history of hypertension, which may reflect differences in the underlying etiology and clinical phenotype of HF across eRAP levels, as well as potential patient selection and residual confounding.

Renal dysfunction is a well-established marker of poor prognosis in HF (14–17) and is closely associated with venous congestion (34). Therefore, we conducted an exploratory mediation analysis. Although lower baseline eGFR was associated with an increased risk of mortality, it did not significantly mediate the association between eRAP and mortality. This finding does not exclude a potential role of renal dysfunction in venous congestion related injury, as baseline eGFR may not adequately capture dynamic changes in renal function. Further prospective studies are needed to clarify these relationships.

## 6 Limitations

Several limitations of this study should be acknowledged. First, this was a single-center retrospective study restricted to patients with available echocardiographic measurements required for eRAP estimation; therefore, selection bias cannot be excluded. The generalizability of our findings may also be limited because the cohort was elderly, predominantly comprised patients with heart failure with preserved ejection fraction (HFpEF), and was characterized by a high proportion of NYHA class IV symptoms and a high mortality rate. Second, eRAP was indirectly estimated from IVC diameter and respiratory collapsibility rather than being directly measured by right heart catheterization. In addition, the timing of echocardiography relative to decongestive therapy was not standardized; therefore, eRAP in this study only reflects the hemodynamic status at a specific time point during hospitalization. Third, although we adjusted for the MAGGIC risk score and further considered other clinically relevant variables in sensitivity analyses, residual and unmeasured confounding cannot be excluded. Finally, only bootstrap internal validation was performed, and independent external validation was not performed. Therefore, the incremental prognostic value of eRAP requires further validation in prospective, multicenter HF cohorts.

## 7 Conclusions

Higher eRAP was associated with an increased risk of all-cause and cardiovascular mortality in patients with HF. It also provided modest incremental prognostic information beyond the MAGGIC risk score. Therefore, eRAP may serve as a complementary indicator to existing risk assessment systems and further improve mortality risk stratification in patients with HF.

## Data Availability

The raw data supporting the conclusions of this article will be made available by the authors, without undue reservation.
