# Supplementary material for: Prognostic value of echocardiography-estimated right atrial pressure beyond the MAGGIC risk score in heart failure patients
Source: Front Cardiovasc Med. 2026 Sep 23;13:1887045. doi: 10.3389/fcvm.2026.1887045 (PMC13642569; doi:10.3389/fcvm.2026.1887045)
Supplement: Supplementary file 1 [file Datasheet1.pdf]

Table S1 Results of Pairwise Comparisons for eRAP

Variables	Normal vs Borderline	Normal vs Elevated	Borderline vs Elevated
SBP (mmHg)	0.035	0.061	1.000
DBP (mmHg)	0.010	0.005	1.000
HR (beats per minute)	0.019	1.000	0.199
BNP	0.068	<0.001	0.155
LVEF	0.288	0.012	0.689
E/e' ratio	1.000	0.022	0.075
MPA Diameter	0.003	<0.001	0.049
HF duration <18 months, n (%)	0.093	0.036	1.000
All-Cause Mortality	0.012	0.007	1.000
CV Death	0.198	0.007	0.653
LVEF Class, n (%)	0.160	0.011	1.000
Hypertension	1.000	0.006	0.128

Abbreviations: SBP Systolic Blood Pressure, DBP Diastolic Blood Pressure, HR Heart Rate, HF Heart Failure, BNP B-type Natriuretic Peptide, LVEF Left Ventricular Ejection Fraction, E/e' ratio Early diastolic mitral inflow velocity to mitral annular tissue velocity ratio, MPA Diameter Main Pulmonary Artery Diameter, MAGGIC Meta-Analysis Global Group in Chronic Heart Failure ,CV cardiovascular

Table S2 Proportional hazards assumption tests

Model	N/events	Variable	χ^2	*P*	PH violation
Model 0[a]	546/232	MAGGIC risk score	0.00	0.995	No
Model 1[b]	546/232	MAGGIC risk score	0.01	0.942	No
		borderline eRAP	0.48	0.488	No
		elevated eRAP	2.78	0.095	No
Model 2[c]	546/232	MAGGIC risk score	0.01	0.939	No
		eRAP, per category increase	2.56	0.110	No
Model 3[d]	546/232	MAGGIC risk score	0.10	0.758	No
		borderline eRAP	0.22	0.639	No
		elevated eRAP	2.07	0.150	No
		BNP	0.77	0.379	No
Model 4[e]	506/207	MAGGIC risk score	0.58	0.445	No
		borderline eRAP	0.31	0.580	No
		elevated eRAP	2.18	0.139	No
		E/e' ratio	0.45	0.503	No
		MPA Diameter	0.98	0.322	No
Model 1 excluding in-hospital deaths	540/226	MAGGIC risk score	0.02	0.898	No
		borderline eRAP	0.76	0.385	No

Model	N/events	Variable	χ^2	*P*	PH violation
		elevated eRAP	1.06	0.304	No

Abbreviations: PH Proportional Hazards, χ^2 chi-square statistic, eRAP echocardiography-estimated Right Atrial Pressure, HR hazard ratio, CI confidence interval, MAGGIC Meta-Analysis Global Group in Chronic Heart Failure, BNP B-type natriuretic peptide, E/e' ratio Early diastolic mitral inflow velocity to mitral annular tissue velocity ratio, MPA main pulmonary artery.
a: MAGGIC risk score
b: eRAP, MAGGIC risk score
c: eRAP (per category increase), MAGGIC risk score
d: eRAP, MAGGIC risk score, BNP
e: eRAP, MAGGIC risk score, E/e' ratio, MPA Diameter

Table S3 Fine-Gray models for the effect of eRAP on secondary endpoints

endpoint	Model	Variables	SHR(95%CI)	*P*
CV Death	Univariate Fine-Gray	eRAP		0.004
		normal eRAP	Reference	
		borderline eRAP	1.36 (0.97-1.90)	0.078
		elevated eRAP	1.82 (1.27-2.61)	0.001
	Model 1[a]	eRAP		0.005
		normal eRAP	Reference	
		borderline eRAP	1.32 (0.94-1.86)	0.107
		elevated eRAP	1.81 (1.26-2.60)	0.001
		MAGGIC risk score	1.11 (1.08-1.14)	<0.001
HF Rehospitalization	Univariate Fine-Gray	eRAP		0.493
		normal eRAP	Reference	
		borderline eRAP	1.03 (0.80-1.33)	0.813
		elevated eRAP	1.18 (0.89-1.56)	0.241
	Model 1[a]	eRAP		0.550
		normal eRAP	Reference	
		borderline eRAP	1.02 (0.79-1.31)	0.898
		elevated eRAP	1.16 (0.88-1.54)	0.288
		MAGGIC risk score	1.02 (1.01-1.04)	0.008

Abbreviations: CV cardiovascular, HF Heart Failure, eRAP echocardiography-estimated Right Atrial Pressure, SHR subdistribution hazard ratio, CI Confidence Interval, MAGGIC Meta-Analysis Global Group in Chronic Heart Failure.
a: eRAP + MAGGIC risk score; Normal eRAP is the reference.

Table S4 Incremental predictive value of eRAP for secondary endpoints

Endpoint	Time	Metric	Model 0[a]	Model 1[b]	*P*
CV Death	6-month	AUC	0.679	0.708	0.016
		Brier score	0.124	0.122	0.012
	1-year	AUC	0.688	0.710	0.020

		Brier score	0.151	0.147	0.004
	2-year	AUC	0.686	0.704	0.028
		Brier score	0.187	0.182	0.004
HF Rehospitalization	6-month	AUC	0.581	0.584	0.596
		Brier score	0.217	0.217	0.244
	1-year	AUC	0.557	0.561	0.308
		Brier score	0.245	0.244	0.180
	2-year	AUC	0.554	0.558	0.312
		Brier score	0.243	0.243	0.236

Abbreviations: CV cardiovascular, HF, Heart failure, AUC Area Under the time-dependent receiver operating characteristic Curve, eRAP echocardiography-estimated Right Atrial Pressure, MAGGIC Meta-Analysis Global Group in Chronic Heart Failure.
a: MAGGIC risk score.
b: eRAP + MAGGIC risk score; Normal eRAP is the reference.
P values were calculated using bootstrap empirical distributions.

Table S5 Cox regression models for sensitivity analyses of the association between eRAP and all-cause mortality

Model	N/events	Variables	HR(95%CI)	*P*	C-Index
Model 0[a]	546/232	MAGGIC risk score	1.11 (1.08-1.13)	<0.001	0.650
Model 1[b]	546/232	Normal eRAP	Reference		0.665
		MAGGIC risk score	1.11 (1.08-1.13)	<0.001	
		Borderline eRAP	1.47 (1.09-1.99)	0.012	
		Elevated eRAP	1.69 (1.22-2.34)	0.001	
Model 2[c]	546/232	MAGGIC risk score	1.11 (1.08-1.13)	<0.001	0.664
		eRAP (per category increase)	1.31 (1.12-1.54)	<0.001	
Model 3[d]	546/232	Normal eRAP	Reference		0.678
		MAGGIC risk score	1.09 (1.07-1.12)	<0.001	
		Borderline eRAP	1.45 (1.07-1.96)	0.016	
		Elevated eRAP	1.59 (1.15-2.20)	0.006	
		BNP	1.00 (1.00-1.00)	<0.001	
Model 4[e]	506/207	Normal eRAP	Reference		0.661
		MAGGIC risk score	1.10 (1.07-1.13)	<0.001	
		Borderline eRAP	1.37 (0.99-1.88)	0.057	
		Elevated eRAP	1.50 (1.05-2.14)	0.025	
		E/e' ratio	1.01 (0.99-1.03)	0.200	
		MPA Diameter	1.03 (1.00-1.07)	0.062	
Model 1 excluding in-hospital deaths	540/226	Normal eRAP	Reference		0.663
		MAGGIC risk score	1.11 (1.08-1.13)	<0.001	
		Borderline eRAP	1.49 (1.10-2.02)	0.009	
		Elevated eRAP	1.58 (1.13-2.20)	0.007	

Abbreviations: eRAP echocardiography-estimated Right Atrial Pressure, HR hazard ratio, CI confidence interval, MAGGIC Meta-Analysis Global Group in Chronic Heart Failure, BNP B-type natriuretic peptide, E/e' ratio Early diastolic mitral inflow velocity to mitral annular tissue velocity ratio, MPA main pulmonary artery.

a: MAGGIC risk score

b: eRAP, MAGGIC risk score

c: eRAP (per category increase), MAGGIC risk score

d: eRAP, MAGGIC risk score, BNP

e: eRAP, MAGGIC risk score, E/e' ratio, MPA Diameter

Table S6 Incremental prognostic performance of eRAP beyond the MAGGIC risk score in sensitivity analyses

Model	-2 log-likelihood	AIC	BIC	C-Index	LR χ^2	*P*	Reference
Model 0[a]	2676.61	2678.61	2682.92	0.650	Reference	Reference	Reference
Model 1[b]	2664.73	2670.73	2683.64	0.665	11.88	0.003	Model 0

Model 2[c]	2665.51	2669.51	2678.12	0.664	11.10	<0.001	Model 0
Model 3[d]	2654.59	2662.59	2679.80	0.678	22.02	<0.001	Model 0
Model 4[e]	2350.03	2360.03	2381.16	0.661	14.51	0.006	Model 0 in the same sample
Model 1 excluding in-hospital deaths	2591.30	2597.30	2610.17	0.663	10.00	0.007	Model 0 in the same sample

Abbreviations: AIC Akaike Information Criterion, BIC Bayesian Information Criterion; C-Index Concordance Index, χ^2 chi-square statistic, eRAP echocardiography-estimated Right Atrial Pressure, HR hazard ratio, CI confidence interval, MAGGIC Meta-Analysis Global Group in Chronic Heart Failure, BNP B-type natriuretic peptide, E/e' ratio Early diastolic mitral inflow velocity to mitral annular tissue velocity ratio, MPA main pulmonary artery.
a: MAGGIC risk score
b: eRAP, MAGGIC risk score
c: eRAP (per category increase), MAGGIC risk score
d: eRAP, MAGGIC risk score, BNP
e: eRAP, MAGGIC risk score, E/e' ratio, MPA Diameter

Table S7 Bootstrap internal validation of the primary model

**Variable**	**Original HR(95% bootstrap CI)**	**Original *P***	**Bootstrap mean HR**	**Bootstrap median HR**	***P*<0.05 proportion**	**Coefficient >0 proportion**
Borderline eRAP	1.47 (1.09-2.00)	0.012	1.49	1.48	0.712	0.995
Elevated eRAP	1.69 (1.23-2.37)	0.001	1.74	1.72	0.891	0.998

Model 1: eRAP, MAGGIC risk score.
MAGGIC Meta-Analysis Global Group in Chronic Heart Failure, eRAP echocardiography-estimated Right Atrial Pressure, HR Hazard Ratio, CI Confidence Interval.
Normal eRAP as the reference.

Table S8 Path models for the exploratory mediation analysis of baseline eGFR

**Adjustment set**	**Path**	**Variable**	**Measure**	**Estimate (95% CI)**	***P***
Unadjusted	eRAP -> eGFR	Borderline vs Normal	Beta	2.55 (-2.68-7.77)	0.341
		Elevated vs Normal	Beta	-0.75 (-6.54-5.04)	0.799
	eRAP -> All-cause mortality	Borderline vs Normal	HR	1.51 (1.12-2.05)	0.007
		Elevated vs Normal	HR	1.74 (1.26-2.41)	<0.001
		eGFR per 10-unit decrease	HR	1.14 (1.09-1.20)	<0.001
	eRAP + eGFR -> All-cause mortality	Borderline vs Normal	HR	1.60 (1.18-2.16)	0.002
		Elevated vs Normal	HR	1.78 (1.29-2.47)	<0.001
		eGFR per 10-unit decrease	HR	1.15 (1.09-1.20)	<0.001
Adjustment set A	eRAP -> eGFR	Borderline vs Normal	Beta	3.05 (-2.09-8.18)	0.245
		Elevated vs Normal	Beta	-1.50 (-7.18-4.18)	0.605
	eRAP -> All-cause mortality	Borderline vs Normal	HR	1.48 (1.10-2.01)	0.010
		Elevated vs Normal	HR	1.90 (1.37-2.63)	<0.001

Adjustment set	Path	Variable	Measure	Estimate (95% CI)	*P*
		eGFR per 10-unit decrease	HR	1.13 (1.07-1.19)	<0.001
	eRAP + eGFR -> All-cause mortality	Borderline vs Normal	HR	1.55 (1.14-2.09)	0.005
		Elevated vs Normal	HR	1.93 (1.39-2.67)	<0.001
		eGFR per 10-unit decrease	HR	1.13 (1.08-1.19)	<0.001
Adjustment set B	eRAP -> eGFR	Borderline vs Normal	Beta	3.56 (-1.39-8.51)	0.159
		Elevated vs Normal	Beta	-1.58 (-7.09-3.93)	0.574
	eRAP -> All-cause mortality	Borderline vs Normal	HR	1.42 (1.05-1.93)	0.024
		Elevated vs Normal	HR	1.79 (1.28-2.51)	<0.001
		eGFR per 10-unit decrease	HR	1.12 (1.06-1.18)	<0.001
	eRAP + eGFR -> All-cause mortality	Borderline vs Normal	HR	1.48 (1.09-2.01)	0.012
		Elevated vs Normal	HR	1.80 (1.28-2.51)	<0.001
		eGFR per 10-unit decrease	HR	1.12 (1.06-1.19)	<0.001

Abbreviations: CI Confidence Interval, HR Hazard Ratio, eRAP echocardiography-estimated Right Atrial Pressure, eGFR, Estimated Glomerular Filtration Rate
set A: eRAP, age, sex, and body mass index.
set B: eRAP, age, sex, body mass index, diabetes mellitus, hypertension, coronary artery disease, chronic obstructive pulmonary disease, smoking status, left ventricular ejection fraction class, and New York Heart Association classification

Table S9 Bootstrap estimates of mediation effects through baseline eGFR

Adjustment set	Comparison	Metric	Estimate (95% CI)	*P*
Unadjusted	Borderline vs Normal	Total effect (log HR)	0.415 (0.110-0.726)	0.004
		Direct effect (log HR)	0.470 (0.158-0.779)	<0.001
		Indirect effect (log HR)	-0.055 (-0.149-0.023)	0.164
		Proportion mediated	-0.133 (-0.666-0.060)	0.168
	Elevated vs Normal	Total effect (log HR)	0.555 (0.226-0.870)	<0.001
		Direct effect (log HR)	0.578 (0.252-0.909)	<0.001
		Indirect effect (log HR)	-0.023 (-0.131-0.069)	0.560
		Proportion mediated	-0.041 (-0.299-0.131)	0.560
Adjustment set A	Borderline vs Normal	Total effect (log HR)	0.395 (0.079-0.687)	0.014
		Direct effect (log HR)	0.436 (0.126-0.736)	0.006
		Indirect effect (log HR)	-0.041 (-0.125-0.031)	0.262
		Proportion mediated	-0.103 (-0.640-0.095)	0.272
	Elevated vs Normal	Total effect (log HR)	0.639 (0.325-0.989)	<0.001
		Direct effect (log HR)	0.655 (0.343-1.016)	<0.001
		Indirect effect (log HR)	-0.016 (-0.114-0.074)	0.688
		Proportion mediated	-0.025 (-0.213-0.117)	0.688

Adjustment set B	Borderline vs Normal	Total effect (log HR)	0.352 (0.056-0.691)	0.024
		Direct effect (log HR)	0.392 (0.084-0.732)	0.008
		Indirect effect (log HR)	-0.041 (-0.117-0.028)	0.284
		Proportion mediated	-0.116 (-0.665-0.114)	0.304
	Elevated vs Normal	Total effect (log HR)	0.584 (0.235-0.972)	0.004
		Direct effect (log HR)	0.586 (0.219-0.972)	0.004
		Indirect effect (log HR)	-0.002 (-0.081-0.072)	0.960
		Proportion mediated	-0.004 (-0.179-0.154)	0.956

Abbreviations: CI Confidence Interval, HR Hazard Ratio, eRAP echocardiography-estimated Right Atrial Pressure, eGFR, Estimated Glomerular Filtration Rate

set A: eRAP, age, sex, and body mass index.

set B: eRAP, age, sex, body mass index, diabetes mellitus, hypertension, coronary artery disease, chronic obstructive pulmonary disease, smoking status, left ventricular ejection fraction class, and New York Heart Association classification

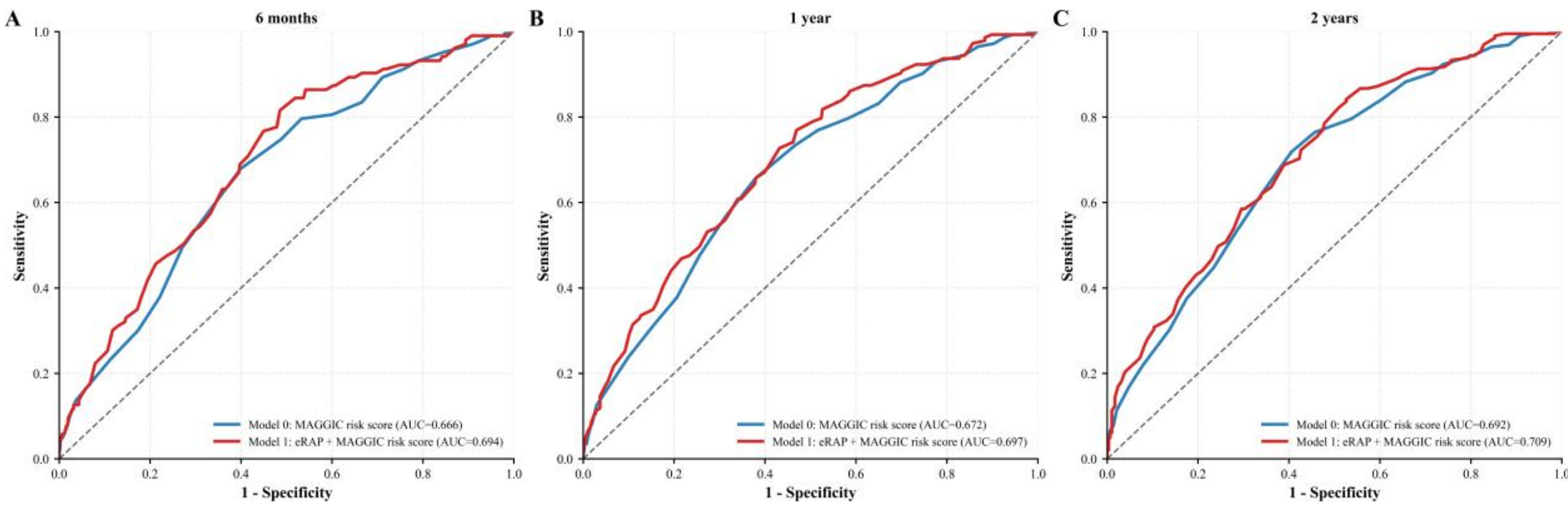

Figure S1 Time-dependent ROC curves of eRAP for all-cause mortality prediction at 6 months, 1 and 2 years

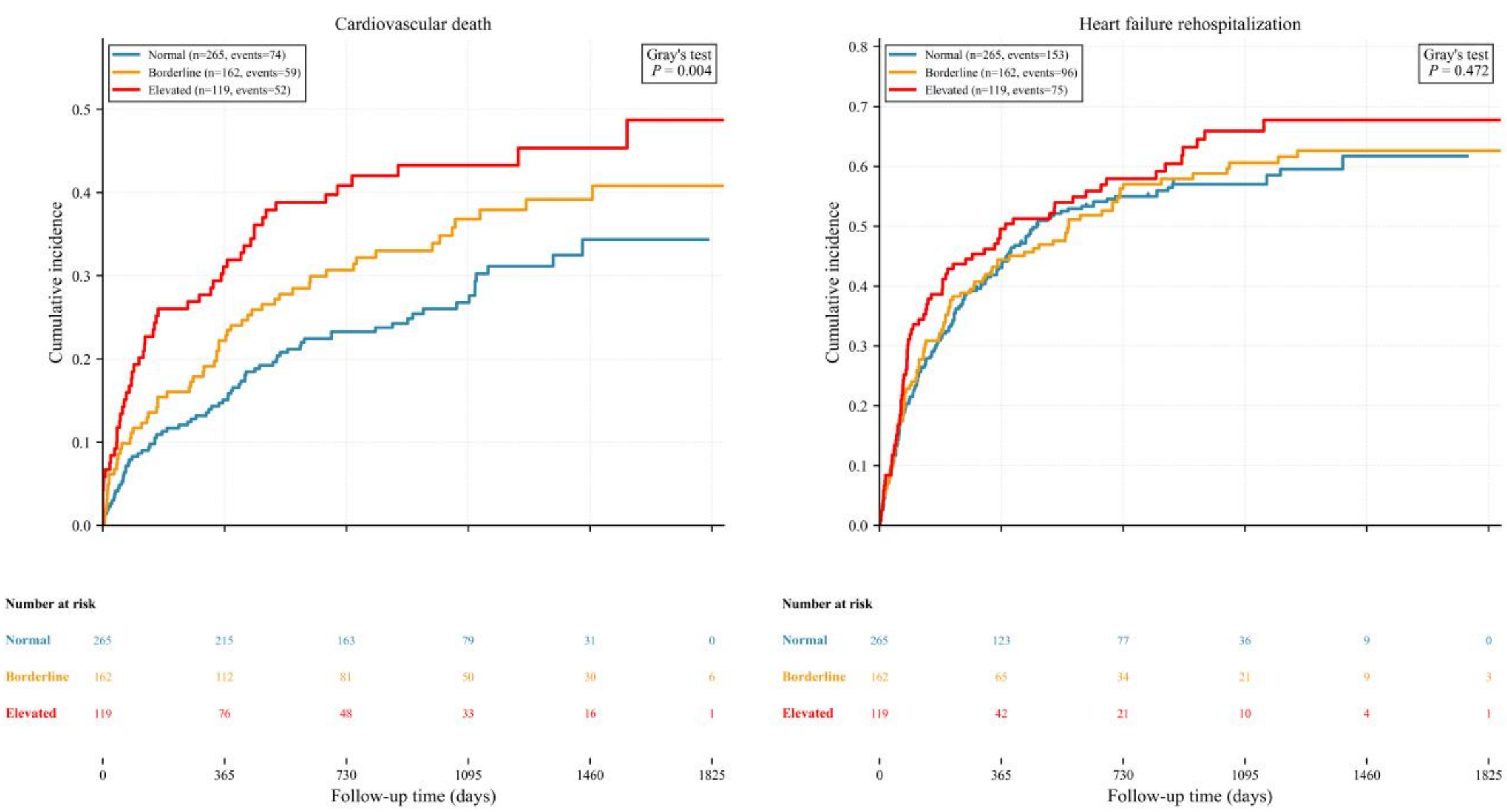

Figure S2 Cumulative incidence of the secondary endpoints by eRAP.

eRAP echocardiography-estimated Right Atrial Pressure
